# Non-invasive assessment of left ventricular contractility by myocardial work index in veno-arterial membrane oxygenation patients: rationale and design of the MIX-ECMO multicentre observational study

**DOI:** 10.3389/fcvm.2024.1399874

**Published:** 2024-05-28

**Authors:** Bálint Károly Lakatos, Zsuzsanna Ladányi, Alexandra Fábián, Réka Ehrenberger, Tímea Turschl, Zsolt Bagyura, Bruno Evrard, David Vandroux, Marine Goudelin, Simon Lindner, Simone Britsch, Daniel Dürschmied, Endre Zima, Gergely Richárd Csikós, Zsolt Túróczi, Ádám Soltész, Endre Németh, Attila Kovács, Ferenc István Édes, Béla Merkely

**Affiliations:** ^1^ Heart and Vascular Center, Semmelweis University, Budapest, Hungary; ^2^ Medical-Surgical ICU, Dupuytren Teaching Hospital, Limoges, France; ^3^ Inserm CIC 1435, Dupuytren Teaching Hospital, Limoges, France; ^4^ Cardiothoracic Intensive Care Unit, Dupuytren University Hospital, Limoges, France; ^5^ Inserm U1094, IRD U270, Univ. Limoges, CHU Limoges, EpiMaCT - Epidemiology of Chronic Diseases in Tropical Zone, Institute of Epidemiology and Tropical Neurology, OmegaHealth, Limoges, France; ^6^ Cardiology, Angiology, Haemostaseology, and Medical Intensive Care, Medical Centre Mannheim, Medical Faculty Mannheim, Heidelberg University, Mannheim, Germany; ^7^ European Center for AngioScience (ECAS), German Center for Cardiovascular Research (DZHK) Partner Site Heidelberg/ Mannheim, and Centre for Cardiovascular Acute Medicine Mannheim (ZKAM), Medical Centre Mannheim, Heidelberg University, Mannheim, Germany; ^8^ Department of Experimental and Surgical Techniques, Semmelweis University, Budapest, Hungary

**Keywords:** extracorporeal membrane oxygenation, echocardiography, myocardial work index, speckle-tracking echocardiography, critical care

## Abstract

**Introduction and aims:**

Veno-arterial extracorporeal membrane oxygenation (VA-ECMO) is an increasingly utilized therapeutic choice in patients with cardiogenic shock, however, high complication rate often counteracts with its beneficial cardiopulmonary effects. The assessment of left ventricular (LV) function in key in the management of this population, however, the most commonly used measures of LV performance are substantially load-dependent. Non-invasive myocardial work is a novel LV functional measure which may overcome this limitation and estimate LV function independent of the significantly altered loading conditions of VA-ECMO therapy. The Usefulness of Myocardial Work IndeX in ExtraCorporeal Membrane Oxygenation Patients (MIX-ECMO) study aims to examine the prognostic role of non-invasive myocardial work in VA-ECMO-supported patients.

**Methods:**

The MIX-ECMO is a multicentric, prospective, observational study. We aim to enroll 110 patients 48–72 h after the initiation of VA-ECMO support. The patients will undergo a detailed echocardiographic examination and a central echocardiography core laboratory will quantify conventional LV functional measures and non-invasive myocardial work parameters. The primary endpoint will be failure to wean at 30 days as a composite of cardiovascular mortality, need for long-term mechanical circulatory support or heart transplantation at 30 days, and besides that other secondary objectives will also be investigated. Detailed clinical data will also be collected to compare LV functional measures to parameters with established prognostic role and also to the Survival After Veno-arterial-ECMO (SAVE) score.

**Conclusions:**

The MIX-ECMO study will be the first to determine if non-invasive myocardial work has added prognostic value in patients receiving VA-ECMO support.

## Introduction

Cardiogenic shock (CS) is the most severe form of acute heart failure, defined as a state in which the heart cannot maintain adequate cardiac output even with markedly increased filling pressures (1). A characteristic feature of CS is systemic organ hypoperfusion, resulting in progressive multi-organ failure, and consequently, poor clinical outcome (2). Despite the constant improvements in pharmacological and supportive therapy, CS has an outstandingly high mortality even in the most advanced healthcare systems worldwide (2).

By implementing a simple concept, e.g., artificial replacement of the function of the cardiopulmonary system, Veno-Arterial Extracorporeal Membrane Oxygenation (VA-ECMO) is a distinct approach in the management of cardiogenic shock (3). VA-ECMO support was utilized in various causes of CS. In patients with CS complicating acute coronary syndrome (ACS), it is hypothesized that VA-ECMO enables to “bypass” that critical period during which substantial myocardial stunning is present, however, randomized clinical trials failed to demonstrate improved prognosis compared to conservative therapy (4, 5). VA-ECMO is also frequently used in CS due to non-ischemic causes (6, 7), or failure to weaning from cardiopulmonary bypass in cardiac surgery patients (8). Early VA-ECMO, as extracorporeal cardiopulmonary resuscitation in cardiac arrest patients is an interesting novel concept, and importantly, initial data seem to support its use (4, 9). Despite the recent studies failed to demonstrate consistent improvement in clinical outcomes compared to the conventional therapy (5, 10), VA-ECMO may provide substantial aid to the cardiopulmonary system leading to favourable outcome in well-selected patients, however, we are still lacking criteria for the most optimal candidacy of the therapy and contraindications (e.g., age ≥ 75 years, severe brain injury, malignancy and other conditions limiting life expectancy) are much more defined (11).

Prolonged VA-ECMO has numerous severe complications. Bleeding events are particularly prevalent in this population, often occurring as a life-threatening complication (12, 13). Beyond haemorrhage, thromboembolic events and the higher incidence of infections are also frequently reported. Therefore, the earliest possible weaning from VA-ECMO is an important therapeutic goal.

The reliable measurement of left ventricular (LV) function is one of the cornerstones to assess the possibility of weaning (14). In clinical practice, echocardiography is the most widely used method for monitoring the LV function. However, even recently developed LV functional measures, such as speckle-tracking echocardiography-derived global longitudinal strain (GLS) are highly load-dependent (15). This load-dependency gains particular importance in certain clinical states, and the significantly deranged loading conditions of VA-ECMO therapy is one of the most relevant ones (16).

Non-invasive myocardial work indices are novel echocardiographic parameters which adjust LV deformation to the instantaneous LV pressure, overcoming the load-sensitivity of the traditional LV functional measures (17). Experimental studies demonstrated that myocardial work indices may be reliable markers of LV contractility in animal models of pressure- or volume-overload-induced heart failure (17). These observations may explain why myocardial work markers were shown to be superior prognostic markers in various clinical settings.

We hypothesize that VA-ECMO patients may also benefit from myocardial work-based LV function evaluation and these novel, non-invasive indices of cardiac performance may be robust markers of patient outcome in this population.

## Study objectives

The “usefulness of Myocardial work IndeX in ExtraCorporeal Membrane Oxygenation patients” (MIX-ECMO) study aims to study the association of non-invasive myocardial work indices with outcome in patients undergoing VA-ECMO therapy.

The primary objective measure is the failure to wean, which consists of different clinical outcomes suggesting the inability to free the patient from MCS:

• Failure to wean: Composite of cardiovascular mortality, need for long-term mechanical circulatory support (MCS) or heart transplantation at 30 days

Besides that, secondary objectives will be also investigated to examine all-cause mortality and certain endpoints of organ-failure:

Secondary outcome measures:

• All-cause mortality at 30 days
• VA-ECMO-free days at 30 days
• Need for renal replacement therapy during intensive care at 30 days
• Successful weaning from mechanical ventilation at 30 days
• Ventilator-free days at 30 days
• Discharge from intensive care unit (ICU) at 30 days
• Discharge from hospital at 30 days

## Study design

MIX-ECMO is an investigator-initiated prospective, multicentre observational study enrolling patients in three European investigational Centres. The study will be conducted in accordance with the Helsinki Declaration, the Good Clinical Practice, and the applicable regulatory requirements.

The study is registered on NIH ClinicalTrials.gov (Identifier: NCT05838937).

## Ethical considerations

The MIX-ECMO study will be conducted in accordance with good clinical practice and the Helsinki Declaration. All regulatory and notified body requirements have been met to perform the study. The MIX-ECMO study has been catalogued and authorized by the Hungarian Medical Research Council under number: BMEÜ/4229-1/2022/EKU. Due to the nature of the examined population, most commonly legally eligible relatives are expected to provide their written informed consent prior to enrolment, which will be collected by certified physicians participating in the study.

## Sample size and patient population

Patients undergoing VA-ECMO (regardless of the indication for the therapy) will be enrolled. The initial plan is to enroll patients within 1 year, with no loss to follow-up. Based on a previous VA-ECMO study investigating echocardiographic predictors of outcome (18), we estimated a 92.2% statistical power to detect a 20% effect with respect to the primary composite outcome, by assuming an 30% weaning failure rate in a sample size of 100 patients. Importantly, the feasibility of speckle-tracking analysis with transoesophageal echocardiography (TOE) was previously reported to be 90% (19). Therefore, we aim to enroll 110 subjects, a sample size which exceeds previous cardiac imaging studies of the field able to demonstrate association of echocardiography measures with outcome (18, 20). Both peripherally and centrally cannulated subjects are candidates for participation. Considering that the two populations are typically quite different in terms of indication for the therapy (ACS- or decompensated chronic heart failure-associated CS vs. cardiac surgery population), a balanced enrollment of the two methods will be facilitated by interim assessment at 50% of the enrollment. All LV venting options are eligible for enrollment, however, if utilized, the mode of LV unloading (central left atrial vent, Impella, transaortic LV pigtail catheter etc.) will also be collected. Notably, LV unloading significantly influences ventricular mechanics (21). Still, due to the highly variable institutional practices in terms of LV unloading, the study protocol does not exclude certain techniques.

The study aims to include a broad spectrum of patients; therefore, the exclusion criteria are mainly technical contraindications of the measurements. Patients 18 years or older on VA-ECMO will be enrolled, if TOE is feasible or the transthoracic echo window also enable sufficient image quality for further analysis. The inclusion and exclusion criteria are listed in Table 1.

**Table 1:** Inclusion and exclusion criteria of the study.

Inclusion criteria	Exclusion criteria
Patients 18 years old or older	Younger than 18 years old
Cardiogenic shock refractory to conventional therapy, requiring VA-ECMO implantation	Written informed consent cannot be collected
Written informed consent (most commonly by a legally eligible relative)	Poor transthoracic window and transesophageal echocardiography is contraindicated (e.g., severe pharyngeal or oesophageal bleeding, extreme coagulation derangement)
	Poor echocardiographic window from both transthoracic and transesophageal approach
	Clinical condition at the time of enrollment which squarely indicates therapy limitation and poor short-term outcome (e.g., established diagnosis of severe cerebral damage, severe hemodynamic instability despite MCS and vasoactive therapy)

**Figure 1:**
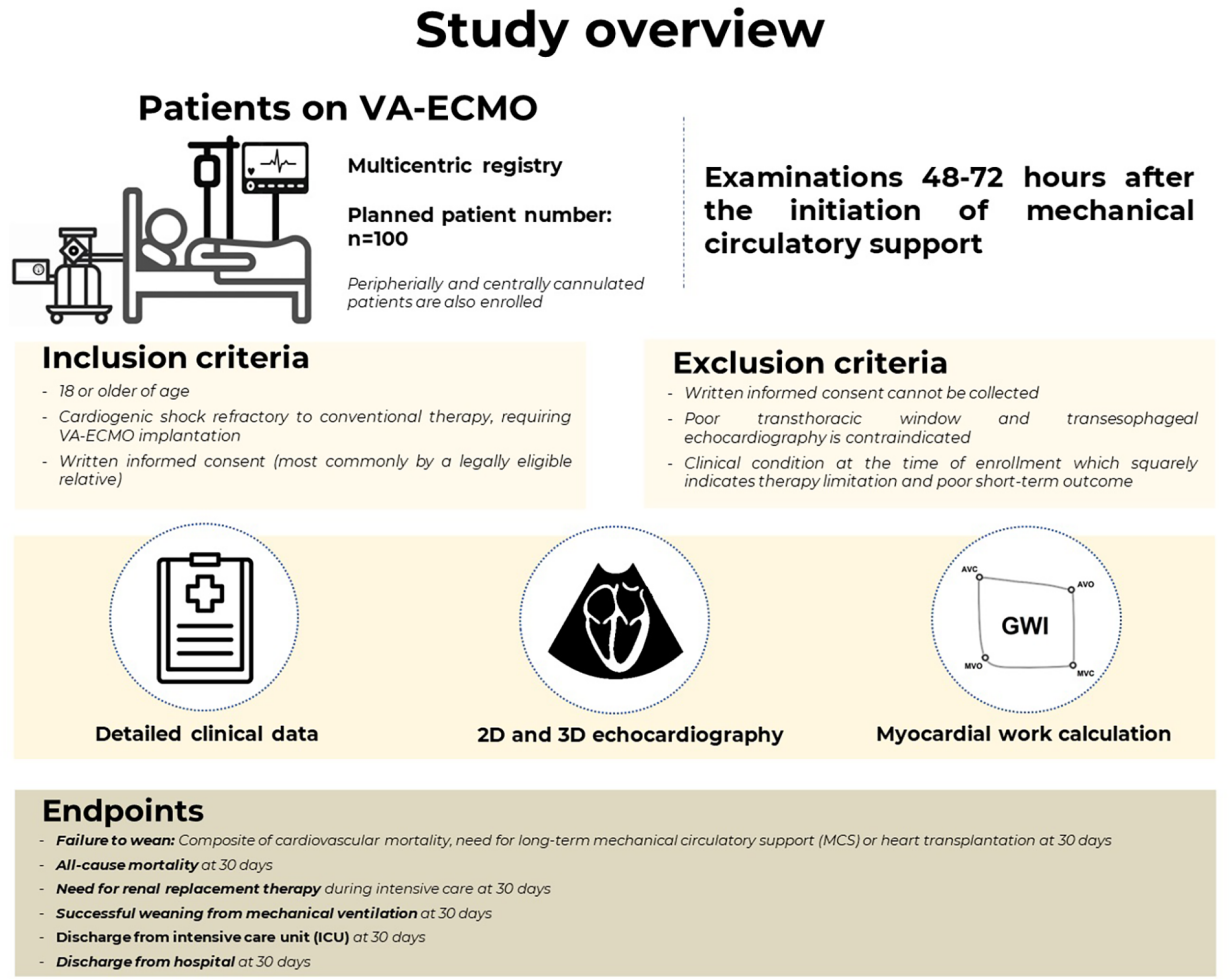
The overview of the study population and the main objectives.

Importantly, enrollment and the study procedures must take place between 48 and 72 h following the initiation of MCS. The main objectives of the study are depicted in Figure 1.

## Statistical analysis

The association of conventional measures of LV function, myocardial work and patient outcome will be assessed by logistic regression, and for certain objectives (ventilator free days, VA-ECMO free days) by linear regression. Despite the limited sample size, a large event rate can be expected in most of the outcome measures, potentially enabling multivariate logistic analyses. Collinearity of variables will be tested at each multivariable model by variance inflation factor (excessive if variance inflation factor >3). To examine the potential added prognostic value of myocardial work to other parameters, the constructed multivariable models will be compared based on Akaike Information Criterion (AIC). Receiver operating characteristic (ROC) curves will be calculated to assess the discriminatory power of the relevant parameters with regards to the study endpoints. Youden index will be used to quantify the optimal cutoff points of each parameter. These cutoff values will be used to dichotomize the study population. Outcomes of the dichotomized groups will be visualized using Kaplan-Meier curves and compared by the log-rank test. All statistical analyses will be performed using SPSS Statistics (IBM Software Group, Armonk NY, USA). A *P*-value of <0.05 will be considered statistically significant.

### Study procedures

The most important collected clinical measures and examinations are summarized in Table 2. All the data will be recorded in a dedicated electronic case report form (eCRF), in line with General Data Protection Regulation (GDPR), in an anonymized manner. The detailed content of the eCRF is depicted in Supplementary Data S2.

**Table 2:** Main collected clinical data.

Collected clinical data
Basic antropometric data
Medical history
Home medication
Indication of ECMO and details of clinical presentation
Parameters of ECMO support and venting
Medical therapy at TOE examination
Parameters of mechanical ventilation
Arterial blood gas data at the time of TOE
Hemodynamic data at the time of TOE
Right heart catheterization data at TOE (optional)
Laboratory data at the day of TOE

ECMO, extracorporeal membrane oxygenation; TOE, transoesophageal echocardiography.

Basic anthropometric data, relevant comorbidities and home medication will be recorded. Based on the given indication for MCS, the details of periprocedural clinical scenario will also be documented.

We will obtain relevant laboratory values at the day of the echocardiographic examination, and the current inotropic and vasopressor support, ventilator settings and most recent arterial blood gas data prior to the acquisition of the echocardiographic images. Right heart catheterization data will also be documented, if available.

### Echocardiographic examination

Echocardiographic acquisitions must be performed between 48 and 72 h following the initiation of MCS. Inotropic and vasopressor support at the time of the examination are based on the clinician's discretion. In order to ensure consistently high image quality, TOE is strongly recommended, however, in patients with good transthoracic echocardiographic window and with relevant concerns regarding TOE (e.g., awake and extubated, clinically relevant oropharyngeal bleeding), transthoracic approach is also acceptable. ECG-gated images and loops will be obtained and at least 3 cardiac cycles will be recorded. The detailed TOE acquisition protocol is depicted in Supplementary Data S1.

3D echocardiographic loops are not mandatory parts of the protocol, however, the acquisition of such datasets are strongly encouraged.

Two sets of acquisitions will be obtained: the first set will be performed with the current MCS minute volume. The second set will be recorded after setting a predefined minute support normalized to the body surface area of the patient (1.1 L/m^2^/min). After changing to the predefined MCS minute volume, only a short (1 min) waiting period is recommended, as the common clinical experience is that biventricular load rapidly changes along with the altered MCS settings.

In order to calculate non-invasive myocardial work indices, systolic and diastolic blood pressure will be recorded at the beginning of both acquisition sets. If the LV does not eject at the time of echocardiographic examination, mean arterial pressure will be used.

Echocardiographic measurements will be performed offline in an Echocardiography Core Laboratory (Semmelweis University, Heart and Vascular Center, Budapest, Hungary) by an experienced operator, blinded to the clinical data and outcome of the patients. All participating sonographers of the Centers will be certified by the Echocardiography Core Laboratory according to the international standards (22).

Cardiac chamber quantification will be based on the most recent guidelines. LV volumes and ejection fraction (EF) will be calculated using the biplane Simpson method. LV longitudinal strain will be measured using dedicated software (TomTec 2D AutoStrain, TomTec Imaging Systems, Unterschleissheim, Germany). LV non-invasive myocardial work indices will be calculated using the exported longitudinal strain data of TomTec and a custom-made software. The principles of myocardial work estimation will correspond with the calculation method of Russell et al. (23).

First, the opening and closure timepoints of the aortic and mitral valves are identified on the echocardiographic loops by visual assessment on a medoesophageal long-axis view. Next, using these temporal reference points, both curves are dissected into four sections (isovolumetric contraction, ejection, isovolumetric relaxation, and diastolic filling), with each section of the strain curve being matched with the corresponding section of the simulated pressure tracing. Due to the different temporal resolution of the datasets, the timestamps of the pressure and strain tracings are normalized in each section. The strain values are interpolated for the timestamps based on the LV pressure recording. The four sections of the recordings are subsequently concatenated, and pressure-strain loops are plotted. The instantaneous power is calculated by multiplying the strain rate (obtained by differentiating the strain curve) and the instantaneous LV pressure.

Global myocardial work index (GWI) is computed by integrating the power from mitral valve closure until mitral valve opening. Work performed during myocardial stretching means energy loss and was defined as negative work in contrast to positive work performed during myocardial shortening. Global constructive work index (GCW) is the sum of global positive work, while global wasted work (GWW) is the sum of negative global work. As a measure of global myocardial efficiency, global work efficiency (GWE) is also calculated as the ratio of the wasted work to the constructive work.

### Assessment of endpoints

**Figure 2:**
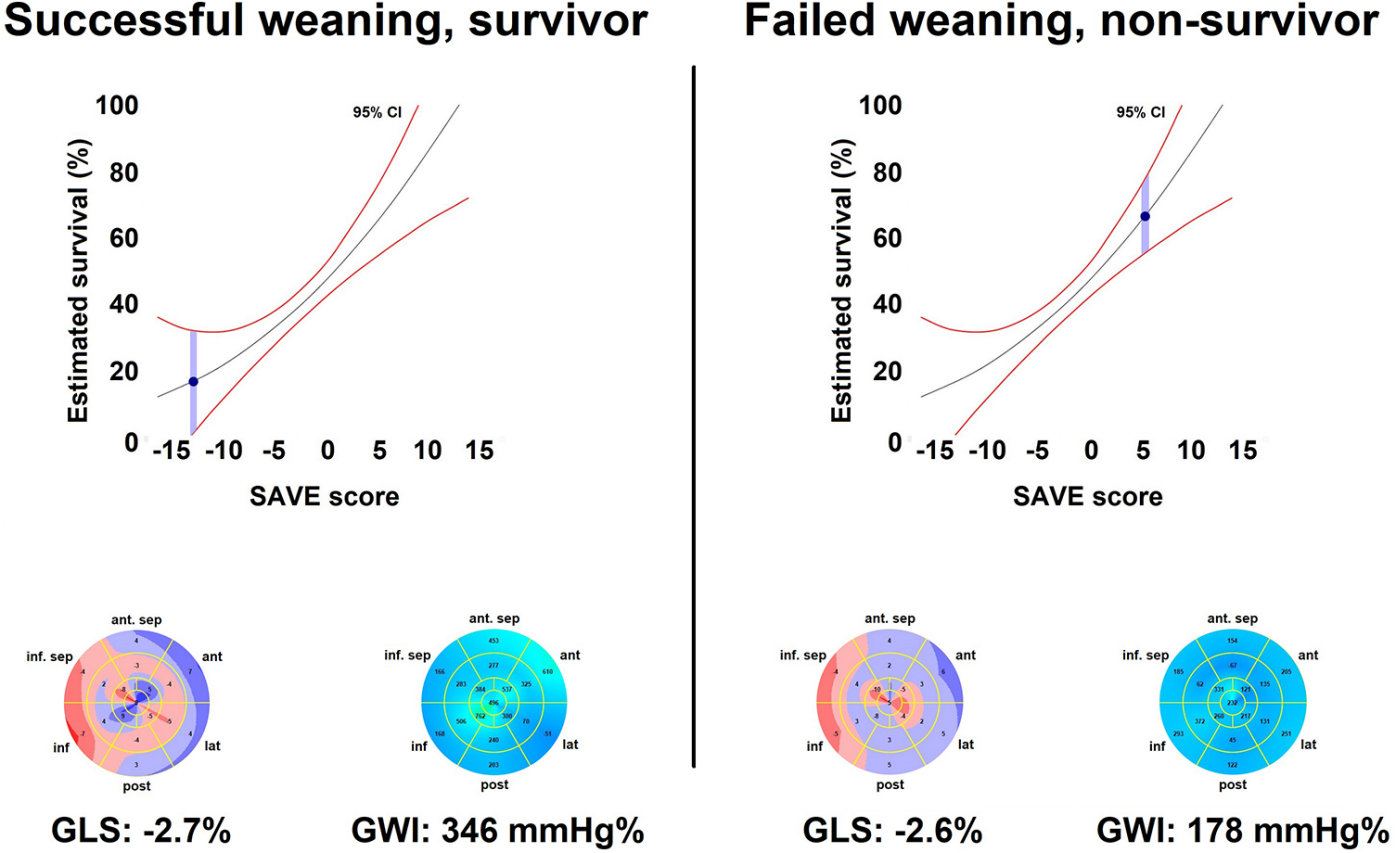
Clinical examples*.* The patient on the left is a typical subject of poor prognosis based on the SAVE score system: an elderly obese male (76 years old) with non-ST elevation myocardial infarction presented at admission with severe heart failure. The patient was admitted with manifest markers of CS. Prior to the catheterization, the patient had cardiac arrest with shockable rhythm, leading to multiple DC shocks and endotracheal intubation. VA-ECMO was initiated, and the patient underwent percutaneous revascularization. On echocardiography, his GWI value was relatively good even on high minute volume support (Supplementary Video S1). Despite the poor expected outcome, the patient was successfully weaned from the mechanical circulatory support, was discharged from the ICU at day 14, and discharged to rehabilitation at day 22. The patient on the right is a young male (35 years old) with decompensated non-ischemic cardiomyopathy. Inotropic support and intravenous diuretic therapy was initiated, however, a slow decline in his circulatory state was observed (INTERMACS profile 2–3). The multidisciplinary team decision was initiation of VA-ECMO, as the patient was eligible for bridging to long-term mechanical circulatory support or heart transplantation. His echocardiography examination is remarkable for practically similar EF and GLS values to the other patient (Supplementary Video S2). Notably, GWI was significantly lower compared to the patient on the left. Despite the complex therapy, the patient had an unfavourable outcome and died in progressive circulatory failure on the 5th day of the VA-ECMO treatment.

The occurrence of study endpoints will be assessed 30 days following the initiation of MCS. An independent, blinded adjudicator will assess the events of the individuals based on the eCRF data. The main objective of the study is to determine if non-invasive myocardial work indices are superior markers of prognosis in this population, compared to the conventional LV functional parameters (such as LV EF, LV GLS or LV outflow tract velocity-time integral). The predictive power of myocardial work indices will also be tested against the Survival After Veno-arterial-ECMO (SAVE) score, which is considered to be the most widely accepted risk stratification score system of this population (24). In Figure 2, two clinical examples are shown: the patient on the left had very low SAVE score, a relatively good GWI value and excellent outcome (Figure 2–left, Supplementary Video S1). The patient on the right had markedly better SAVE score, however, his GWI value was significantly worse and eventually had unfavourable outcome (Figure 2–right, Supplementary Video S2).

## Discussion

VA-ECMO support has become an essential tool for the therapy of cardiac arrest and severe refractory CS in many cardiothoracic centres and intensive care units worldwide (25). Still, the initiation of VA-ECMO is a double-edged sword: the improvement of the systemic organ perfusion comes inherently with the cost of a markedly increased complication rate (3).

Several score systems were introduced to predict survival in VA-ECMO support. The most commonly used one is the SAVE score, based on the clinical characteristics and outcome data of an exceptionally large cohort of almost 4,000 patients (24). Beyond the underlying aetiology and basic anthropometric data, several markers of the macro- and microcirculation are used to estimate survival, which highlights the fact the multiple factors influence the outcome in this population, with a special importance on the metabolic derangement prior to the initiation of MCS. Notably, external validation studies confirmed systematic underestimation of survival (26). Other score systems were also introduced, however, recent studies identified Simplified Acute Physiology Score II (SAPS II) and Sequential Organ Failure Assessment (SOFA) as strong discriminators of survivors and non-survivors, showing that the current VA-ECMO-specific scores do not necessarily outperform “general” critical care risk calculators in real-life (27, 28). These observations suggest that the implementation of cardiac functional measures may provide added prognostic value in this highly specific population. Importantly, “very short term” survival is strongly determined by the initial hemodynamic derangement (very low pH, high lactate levels at the “plateau” of shock) and less by the LV function, therefore, in MIX-ECMO the echocardiographic examination takes place 48–72 h after the initiation of MCS (29).

Still, serial assessment of LV structure and function are cornerstones in the management of patients with CS. LV EF is the mainstay parameter of LV performance, however, it is known to be strongly influenced by various hemodynamic factors, such as loading conditions and chamber geometry (15). Therefore, LV EF cannot be perceived as a marker of contractility, and acts more like an integrative measure of LV performance. This is strongly underpinned by the fact, that the majority of patients admitted with acute heart failure have preserved LV EF, and a considerable proportion of CS admissions are presented with preserved LV EF (30).

Recently, other LV functional measures emerged as potential candidates to overcome this issue. Tissue Doppler Imaging-based myocardial velocities and speckle-tracking-derived GLS was shown to be superior markers of LV systolic function with established prognostic value in acute heart failure (30). Nevertheless, even such measures of the LV performance are shown to be highly load-dependent, limiting their comparability in a patient-to-patient basis.

VA-ECMO significantly alters LV load, which hinders the actual contractile state of the chamber: LV unloading may decrease the value of traditional functional measures, while the concomitant increase in LV afterload further deteriorates LV ejection and may obscure an otherwise maintained LV inotropy (21). Notably, methods of LV unloading, such as concomitant Impella support, intra-aortic balloon pump or direct surgical venting have markedly different effect on LV load, further complicating the interpretation of LV function in a VA-ECMO-supported patient (21, 31). Generally, pressure-volume loops are shifted to the left resulting in lower LV volumes and pressures, while Impella also results in significant shortening of the isovolumetric phases. Moreover, LV venting (transaortic pigtail, LA cannula) does not modify LV afterload, on the other hand, Impella and intraortic balloon pump unloads the LV by decreasing the afterload. As a sum of these influencing factors, LV performance during MCS barely represents ventricular function after weaning. Analysis of the Extracorporeal Life Support Organization (ELSO) registry demonstrated that systolic blood pressure during MCS has incremental prognostic value over the SAVE score, showing that pressures generated by the LV are of great importance (32). Therefore, we hypothesize that the integrative assessment of LV function and concomitant pressures may overcome the limitation of conventional LV functional measurements. Similarly, to this concept, a recent study showed that right ventricular GLS-based measures of right ventricle-pulmonary artery coupling are associated with prognosis in VA-ECMO patients (20).

Myocardial work indices examine LV deformation in the context of LV pressures: by the measurement of LV longitudinal strain and the estimation of the LV pressure curve using a simple blood pressure measurement, pressure-strain curves will be generated. Experimental data suggest that myocardial work index may be a marker of contractility in animal models of pressure- or volume-overload-induced heart failure (17). Growing evidence indicates that myocardial work has as added prognostic value in a wide variety of diseases, especially in states of altered loading conditions, such as valvular diseases (33, 34). Nevertheless, its potential role in the MCS population is still waiting to be tested in clinical studies.

## Conclusions

Despite the constantly increasing MCS utilization worldwide, data are still scarce regarding the factors of successful weaning and survival. The LV contractile state may be a key factor of favourable clinical outcome, however, we were lacking parameters which may reliably estimate it, especially in the case of markedly deranged loading conditions, such as VA-ECMO therapy. Myocardial work parameters may overcome this issue by giving a load-independent measure of LV function. The MIX-ECMO will be the first multicentric study to investigate if myocardial work may have an added prognostic value in VA-ECMO patients.

## Supplementary material

Supplementary Data S1 Detailed echo acquisition protocol.

Supplementary Data S2 Detailed content of the electronic case report form.

Supplementary Video S1 Midoesophageal two-chamber view of the patient on the left.

Supplementary Video S2 Midoesophageal two-chamber view of the patient on the right.
