# Supplementary material for: Non-invasive assessment of left ventricular contractility by myocardial work index in veno-arterial membrane oxygenation patients: rationale and design of the MIX-ECMO multicentre observational study
Source: Front Cardiovasc Med. 2024 May 28;11:1399874. doi: 10.3389/fcvm.2024.1399874 (PMC11165188; doi:10.3389/fcvm.2024.1399874)
Supplement: Supplementary Data S1 — Detailed echo acquisition protocol. [file Datasheet1.docx]

**The usefulness of Myocardial work IndeX in ExtraCorporeal Membrane Oxygenation patients (MIX-ECMO) – echo protocol**

***Principles***

- The examination takes place 48-72 hours following the initiation of mechanical circulatory support
- Inotropic and vasopressor support at the time of the examination are based on the clinician’s discretion
- The ***first set*** of acquisitions are performed at the actual ECMO flow
- The ***second set*** of acquisitions are performed at a standard ECMO flow (1.1 L/min/m^2^) adjusted to the body surface area
- Arterial blood pressures are recorded at the beginning of both datasets
  - in the case of left ventricular (LV) ejection, systolic and diasolic pressure as well
  - in the case of non-ejecting LV, only mean arterial pressure
- *Ultrasound system with* TOE transducer (preferably 3D-capable, but not mandatory)
- *ECG-gated acquisitions*, at least 3 heart cycles obtained per required loop
- Acquisitions are saved in an anonymous manner in DICOM format, and uploaded to an online interface for central Echocardiographic Laboratory reading

***Required loops***

- **midesophageal four-chamber view (0°)**
  - LV-focused loop
  - RV-focused loop
  - Color Doppler of the mitral valve
  - Color Doppler of the tricuspid valve
  - CW-Doppler interrogation of the tricuspid regurgitant jet, if possible
  - CW-Doppler interrogation of the mitral regurgitant jet, if possible
- **midesophageal two-chamber view (90°)**
  - LV-focused loop
  - Color Doppler of the mitral valve
- **midesophageal long-axis view (120-130°)**
  - LV-focused loop
  - Color Doppler of the mitral valve
  - Color Doppler of the aortic valve
- **transgastric short-axis view at the level of the papillary muscles**
  - LV-focused loop
- **deep transgastric long-axis view**
  - PW-Doppler interrogation of the LV outflow tract
  - In the case of prosthetic aortic valve, CW-Doppler interrogation of the aortic valve
- **deep transgastric RV inflow-outflow view**
  - Color Doppler of the pulmonary valve
  - PW-Doppler interrogation of the RV outflow tract
- **3D LV- and RV-focused acquisitions, *if possible***
- If certain Doppler loops can be obtained from other views with a substantially better quality, the acquisition of these loops are also encouraged
- In the case of hemodynamically significant valvular disease, additional loops for the quantitative assessment (e.g. EROA and regurgitant volume of a valvular insufficiency) is also recommended
