# Supplementary material for: Non-invasive assessment of left ventricular contractility by myocardial work index in veno-arterial membrane oxygenation patients: rationale and design of the MIX-ECMO multicentre observational study
Source: Front Cardiovasc Med. 2024 May 28;11:1399874. doi: 10.3389/fcvm.2024.1399874 (PMC11165188; doi:10.3389/fcvm.2024.1399874)
Supplement: Supplementary Data S2 — Detailed content of the electronic case report form. [file Datasheet2.docx]

| **SECTION** | **Data** | **Conditional data** |
| --- | --- | --- |
| **Basic anthropometric data** | Date of birth |  |
|  | Date of admission |  |
|  | Age |  |
|  | Sex |  |
|  | Height |  |
|  | Weight |  |
|  | Body surface area |  |
| **Comorbidities** | Hypertension |  |
|  | Diabetes mellitus |  |
|  | Chronic kidney disease |  |
|  | Chronic obstructive pulmonary disease |  |
|  | Ischaemic heart disease | Previous PCI/CABG |
|  |  | Previous myocardial infarction |
|  | Heart failure (regardless of ejection fraction) |  |
|  | Last ejection fraction before MCS (if available) |  |
|  | Stroke/TIA |  |
|  | Peripherial artery disease |  |
|  | Malignancy |  |
|  | Pulmonary embolism |  |
|  | Atrial fibrillation/fluttern |  |
|  | Liver failure |  |
|  | Congenital heart disease | Type of congential heart disease |
|  | Pacemaker: | VVI |
|  |  | AAI |
|  |  | DDD |
|  |  | VDD |
|  |  | CRT |
|  |  | ICD |
|  |  | none |
|  | Other (comorbidity) |  |
| **Home medication** | ACEi/ARB |  |
|  | ARNI |  |
|  | Beta-blocker |  |
|  | MRA |  |
|  | SGLT2i |  |
|  | Diuretics |  |
|  | Ivabradine |  |
|  | CCB |  |
|  | VKA |  |
|  | DOAC |  |
|  | Aspirin |  |
|  | Clopidogrel |  |
|  | Prasugrel |  |
|  | Ticagrelor |  |

| **Indication of VA-ECMO** | Pulse pressure pre-ECMO ≤20 mmHg? |  |
| --- | --- | --- |
|  | Diastolic blood pressure pre-ECMO≤40 mmHg? |  |
|  | HCO3 pre-ECMO ≤15 mmHg? |  |
|  | Central nervous system dysfunction? |  |
|  | ***If ACS/acute chest syndrome:*** |  |
|  | Clinical presentation: | CCS/UA |
|  |  | STEMI |
|  |  | NSTEMI |
|  | Culprit lesion: | LM |
|  |  | LAD |
|  |  | CX |
|  |  | RCA |
|  |  | IM |
|  | Time to admission |  |
|  | Door-to-cath lab time |  |
|  | Needle-to-MCS time |  |
|  | prehospital/pre-MCS VT/VF |  |
|  | CPR prior to MCS: | Length of CPR |
|  | Mechanical ventilation prior to MCS: | Initiation time of mechanical ventilation |
|  | GCS score prior to sedation |  |
|  | ***If cardiac surgery - unsuccessful CPB weaning:*** |  |
|  | Indication for surgery |  |
|  | CABG |  |
|  | AVR |  |
|  | MVR |  |
|  | TVR |  |
|  | PVR |  |
|  | HTX |  |
|  | Aortic surgery |  |
|  | Brief description of procedure |  |
|  | Length of surgery |  |
|  | CPB time |  |
|  | Aortic clamping time |  |
|  | GCS score prior to sedation |  |
|  | Initiation of mechanical ventilation |  |
|  | ***If heart failure - low-output syndrome:*** |  |
|  | VT/VF prior to MCS |  |
|  | CPR length prior to MCS |  |
|  | Myocarditis? |  |
|  | INTERMACS stage prior to MCS |  |
|  | Mechanical ventilation prior to MCS: | Initiation time of mechanical ventilation |
|  | GCS score prior to sedation |  |

| **VA-ECMO support parameters** | Cannulation: | peripherial |
| --- | --- | --- |
|  |  | central |
|  | MCS minute volume at initiation |  |
|  | MCS minute volume at TOE examination |  |
|  | LV venting: | Transaortic catheter venting |
|  |  | surgical LA cannula |
|  |  | Impella |
|  |  | Impella minute volume |
|  |  | none |
|  | IABP |  |
| **Therapy at TOE examinaniation** | Noradrenaline |  |
|  | Vasopressin |  |
|  | Dobutamine |  |
|  | Milrinone |  |
|  | Levosimendan |  |
|  | Furosemide (/24h) |  |
|  | Na-heparine |  |
|  | Temporary pacemaker |  |
|  | Renal replacement therapy |  |
|  | Last 24h urine output |  |
|  | Temperature |  |
| **Ventilation parameters** | Mode of ventilation |  |
|  | VT |  |
|  | PEEP |  |
|  | FiO2 |  |
|  | Ppeak |  |
|  | Pmean |  |
|  | RR |  |
|  | Pplat (if measured) |  |
|  | Ptranspulm (if measured) |  |
| **Arterial blood gas** | pO2 |  |
|  | pCO2 |  |
|  | pH |  |
|  | Lac |  |
|  | HCO3st |  |
|  | BE |  |
| **Hemodynamic data** | SBP |  |
|  | DBP |  |
|  | CVP |  |
|  | HR |  |
|  | Rhythm: | Sinus |
|  |  | Atrial fibrillation/fluttern |
|  |  | Pacemaker rhythm |

| **Right heart catheterization (if available)** | CVP |  |
| --- | --- | --- |
|  | RVSP |  |
|  | RVDP |  |
|  | PASP |  |
|  | PADP |  |
|  | PAWP |  |
|  | CO |  |
|  | PVR |  |
|  | SVR |  |
| **Laboratory data** | WBC |  |
|  | Neutrophil% |  |
|  | Lymphocyte% |  |
|  | Hemoglobin |  |
|  | Hematocrit |  |
|  | Thrombocyte |  |
|  | Na |  |
|  | K |  |
|  | Cl |  |
|  | Ca |  |
|  | Mg |  |
|  | P |  |
|  | Creatinine |  |
|  | BUN |  |
|  | ALT |  |
|  | AST |  |
|  | GGT |  |
|  | ALP |  |
|  | LDH |  |
|  | Total bilirubin |  |
|  | hs-TroponinT |  |
|  | NT-proBNP |  |
|  | CRP |  |
|  | PCT |  |
|  | CK |  |
|  | CK-MB |  |
